# Reduction of Radiation Doses to Patients and Staff During Endoscopic Retrograde Cholangiopancreatography

**DOI:** 10.4103/1319-3767.74456

**Published:** 2011

**Authors:** Abdelmoneim Sulieman, Georgios Paroutoglou, Andreas Kapsoritakis, Anargeyros Kapatenakis, Spiros Potamianos, Marianna Vlychou, Kiki Theodorou

**Affiliations:** 1 Department of Medical Physics, University of Thessaly, University Hospital of Larissa, P.O.Box 1425, Larissa 41110, Greece; 2 Public Gastroenterology Department, University of Thessaly, University Hospital of Larissa, P.O.Box 1425, Larissa 41110, Greece; 3 University Gastroenterology Department, University of Thessaly, University Hospital of Larissa, P.O.Box 1425, Larissa 41110, Greece; 4 Radiology Department, University of Thessaly, University Hospital of Larissa, P.O.Box 1425, Larissa 41110, Greece; 5 Department of Medical Physics, College of Medical radiologic Science, Sudan University of Science and Technology, Khartoum, Sudan

**Keywords:** ERCP, radiation risk, staff exposure

## Abstract

**Background/Aim:**

Endoscopic retrograde cholangiopancreatography (ERCP) is associated with a considerable radiation exposure for patients and staff. While optimization of the radiation dose is recommended, few studies have been published. The purpose of this study has been to measure patient and staff radiation dose, to estimate the effective dose and radiation risk using digital fluoroscopic images. Entrance skin dose (ESD), organ and effective doses were estimated for patients and staff.

**Materials and Methods:**

Fifty-seven patients were studied using digital X-ray machine and thermoluminescent dosimeters (TLD) to measure ESD at different body sites. Organ and surface dose to specific radiosensitive organs was carried out. The mean, median, minimum, third quartile and the maximum values are presented due to the asymmetry in data distribution.

**Results:**

The mean ESD, exit and thyroid surface dose were estimated to be 75.6 mGy, 3.22 mGy and 0.80 mGy, respectively. The mean effective dose for both gastroenterologist and assistant is 0.01 mSv. The mean patient effective dose was 4.16 mSv, and the cancer risk per procedure was estimated to be 2 × 10^-5^

**Conclusion:**

ERCP with fluoroscopic technique demonstrate improved dose reduction, compared to the conventional radiographic based technique, reducing the surface dose by a factor of 2, without compromising the diagnostic findings. The radiation absorbed doses to the different organs and effective doses are relatively low.

The population exposure from medical X-rays contributes approximately 14% of the average annual population dose. More than 95% of human exposure to man-made ionizing radiation results from diagnostic and interventional radiology.[1,2] Ionizing radiation is a well-established risk factor for cancer. Since medical exposure has been justified, due to the potential benefit to the patient, there are no prescribed dose limits to the patients; however, the principles of radiation protection have to be followed.[1–4]

The International Commission on Radiological Protection (ICRP)[5,6] has recommended dose limits for occupational exposure in order to reduce the probability of cancer and to prevent the tissue reaction effects. Examiners can reduce the occupational exposure to radiation by using the principles based on distance, time, and shielding.

The operator selects equipment and methods to ensure that for each medical exposure, the dose of ionizing radiation to the individual undergoing the exposure is as low as reasonably achievable (ALARA) and consistent with the intended diagnostic or therapeutic purpose.[7] However, despite the fact that endoscopic retrograde cholangiopancreatography (ERCP) requires fluoroscopic and radiographic exposures, which impose radiation risks to patients and staff, the exposure of gastroenterologists and patients to ionizing radiation and the associated potential cancer risk have been assessed in only a few studies.[8–16]

The purpose of this study has been to determine the occupational doses of ionizing radiation and to estimate the related risks to the patients and staff at gastroenterology department.

## MATERIALS AND METHODS

### Patient dose measurement

Fifty-seven patients underwent therapeutic ERCP. This prospective study was conductedat Larissa University hospital, Greece. Clinical indications for the investigation of ERCP are presented in Table 1. The ethics and research committee approved the study and a written consent was obtained from all patients prior to the procedure. Thermoluminescent dosemeters (TLDs) were packed on a thin envelope made of transparent plastic foil, to protect them from any contamination. Each envelope contained three TLDs. Three envelopes were used to measure the ESD, exit dose and thyroid surface dose accurately for each patient. It was important to determine the exit dose as it reflected the transmission of the radiation and the radiation dose to the interior organs. During the procedure, the TLDs were kept in the required positions (entrance of the radiation [at intersection point of the X-ray beam axis with the entrance surface of the patient], exit of radiation and thyroid gland) and were stuck in place with adhesive tape. The operators performed the investigations as their daily practice with a protocol that is designed to minimize patient and examiner exposure.[17]

**Table 1:** ERCP indications

Indications	Total	%
CBD stones	29	50.9
Post operation leakage	3	5.2
Cholangitis	6	10.5
Malignancy	8	14.0
Benign CBD stricture	1	1.8
Stent removal or exchange	5	8.8
Pancreatitis	5	8.8
Total	57	100

ERCP: Endoscopic retrograde cholangiopancreatography, CBD: common bile duct

The data recorded for all procedures included patient body characteristics (age, sex, height, weight and body mass index (BMI) (weight/height ^2^), tube voltage (kV), tube load (mAs), and fluoroscopic data: kV, tube current (mA), total screening time, clinical indication, start and end time.

### Staff dose measurement

Two experienced gastroenterologists (more than three thousands procedures) performed all the procedures. Regarding gastroenterologist, the radiation dose was monitored using three TLDs packed on a thin envelope made of transparent plastic foil and were stuck in place with adhesive tape, at four sites: the forehead, thyroid, chest, and left hand. The staff used a 0.25 mm lead equivalent thick apron, full wrap-around protection (Dr. Goos-Suprema GmbH, Heidelberg, Germany). The assistant used 0.50 mm lead equivalent thickness, frontal protection (Rheix-srl, Milan, Italy). TLDs were packed on a thin envelope made of transparent plastic foil, to protect them from any contamination. Each envelope contained three TLDs. TLDs were attached outside the lead apron at the chest level and at the left hand of the assistant. Neither a protective eyeglass nor thyroid collar were worn by either of the staff. The examiner radiation dose in gastroenterology departments is routinely monitored by TLD dosemeters.

During the procedure, the first examiner stood on the right side of the typical position of the patient. A lead barrier (100 × 60 cm^2^) of 0.50 mm lead equivalent was placed on the side of the gastroenterologsit to reduce radiation scatter to the examiners standing to the side of the fluoroscopy couch.

**Figure 1:**
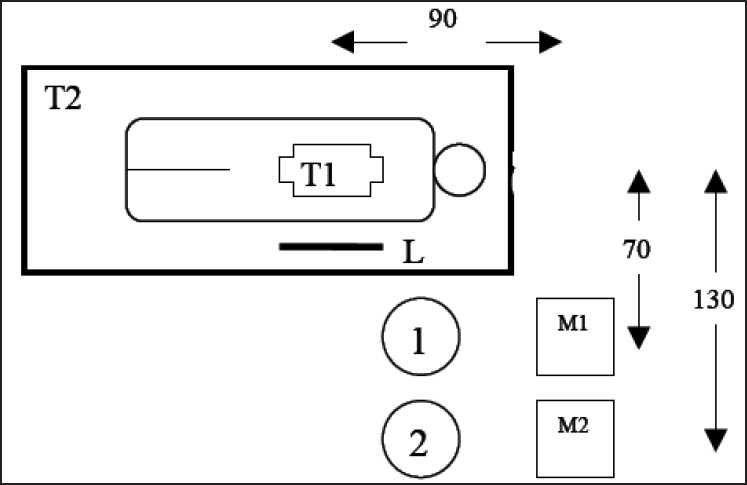
Patient setup, lead apron and examiners positions during ERCP examination. 1. Gastroenterologist; 2. assistant gastroenterologist; M1., endoscopic monitor; M2. fluoroscopic monitor. T1 X ray tube. T2,Table, and L lead apron

The assistant, who controlled the radiation exposure, stood on the right side of the first examiner. All procedures were performed with the examiners at the same locations [Figure 1]. The nurses remained outside the X-ray room during the exposure; therefore, there was no need for radiation dose measurements.

### ERCP technique

ERCP was performed with a duodenoscope (Olympus, exera CLE 145(Olympus Medical System Corp, Japan)). The patient was placed on an X-ray couch in the left anterior oblique position with right leg flexion.

During the procedure, radiographic and fluoroscopic images were obtained after injection of contrast medium. Since the contrast medium normally remained in the biliary tree for several minutes following removal of the duodenoscope, a post procedure anteroposterior projection was also obtained, if required, for further evaluation of the stent placement or residual stones.

### TLD measurements

Entrance surface dose (ESD) was made by attaching a sachet containing four thermoluminiscent dosemeters (TLDs) to the patients’ skin on the central axis of the X-ray beam. The lithium fluoride TLD chips (TLD-100) were used for patient dose measurements while calcium fluoride was used for staff dose measurements for their numerous advantages.[18] The read-out of TLD dosemeter was made using a manual TLD reader (Harshaw 3500, Solon, USA). The overall system performance was checked before any reading session. The read-out was at a 10°C preheat temperature and the signal was acquired from 100 to 280°C with heating rate of 100°C s-1. Prior to each irradiation, all dosemeters were annealed (as recommended by the manufacturer) in annealing oven (TLDO, PTW, Freiburg, Germany) at 400°C for 1 h, followed by fan forced cool down to 1000°C which was held for 2 h in order to optimize its characteristics.

### Radiographic equipment

This study was performed using an overcouch X-Ray (Philips Diagnost 93) fluoroscopy machine. Total filtration of the X-ray beam was 4.0 mm Al. The machine had an option of selecting pre-programmed exposure factors based on the type of examinations performed. Personnel involved in operating the machine could also manually change the pre-programmed exposure factors. The machine also had the option to capture the last fluoroscopic image.

### Estimation of absorbed organ doses and effective doses

ESD was used to estimate the organ equivalent dose (*H*) using software provided by the National Radiological Protection Board (NRPB-SR262).[19] It contains the results of modelling conditions of exposure relevant to 68 common radiographic views. For each view, normalized doses are presented for 26 organs or tissues.

However, as specific projections were not available for ERCP in the aforementioned software, organ doses (mGy) were obtained from the average value of the conversion factors for the most similar projections, i.e PA kidney, stomach and oblique duodenum views.

The organ equivalent dose (*mSv*) is given by:

(1) $$\underset{T}{H}\;=\;\sum_{R}w_{R}.D_{T,R}$$

Where D_T, R_ is the mean absorbed dose to tissue (T) from radiation (R) and w_R_ is the radiation-weighting factor.[5,6]

Effective dose (*E, mSv*) is a quantity that has been introduced to give an indication of risk from partial or non-uniform exposure in terms of the equivalent whole body exposure which gives the same risk.[4,5] :

(2) $$E\;=\;\sum_{T}w_{T}.H_{T}$$

Where *H_T_* is the equivalent dose to tissue *T*.

The examiners’ *E* can be estimated by using the following formulae[20,21]:

(3) $$E\;=\;0.06\left(H_{OS}\;-\;H_{U}\right)+H_{U}$$

Where H_OS_ is the dose measured by the dosemeter at the neck (shallow depth) and H_U_ is the dose measured by the dosemeter under the apron at waist level (deep). In a case of single dosemeter worn at collar level, usually, H_U_ = 0.01 H_OS_, therefore, the effective dose can be approximated as:

(4) $$E\;=\;0.07H_{OS}$$

### Cancer risk estimation

The risk (R_T_) of developing cancer in a particular organ (T) following ERCP after irradiation was estimated by multiplying the mean organ equivalent (H_T_) dose with the risk coefficients (f_T_) obtained from ICRP.[4,5]

(5) $$R_{T}\;=\;H_{T}.f_{T}$$

The overall lifetime mortality risk (*R*) per procedure resulting from cancer/heritable was determined by multiplying the effective dose (*E*) by the risk factor (*f*).

(6) $$R\;=\;E\;.f\;=\;\sum R_{T}$$

The risk of genetic effects in future generations was obtained by multiplying the mean dose to the ovaries by the risk factor.[3–6,22,23]

### Statistical analysis used

All values of radiation dose were expressed as mean, median, minimum, third quartile and the maximum values are presented due to the asymmetry in data distribution.

## RESULTS

A total of 57 ERCP procedures were performed over five months (32 males and 25 females). The total successful procedures were for 54 patients (94.7%). Patients’ body characteristics, screening time, number of radiographic and fluoroscopic images, and the procedure duration are presented in Table 2. Considerable variations were observed among patient populations in terms of radiation dose, and fluoroscopic time [Tables 1–3]. These variations are due to the different indications, patient characteristics and clinical indications [Table 1].

**Table 2:** Patient body characteristics (age, height, BMI and weight), screening time and number of radiographic and fl uoroscopic images. (mean and the range in the parentheses)

Age group	n	Patient age (year)	Height (cm)	Weight (Kg)	BMI (Kg/m^2^)	Screening time (min)	No. of radiographic images	Procedure duration (min)
All	57	65.8 (26-91)	163.5 (149-186)	74.6 (47-110)	27.3 (17.9-42.9)	2.9 (0.3-12.3)	2.6 (1-6)	27.5 (15-55)
Males	32	64.9 (26-86)	165.4 (149-186)	74.9 (47-106)	27.5 (17.9-40.6)	2.6 (0.3-12.3)	2.1 (1-5)	25 (15-50)
Females	25	65.8 (27-91)	163.6 (150-185)	74.6 (50-110)	26.6 (18.6-42.9)	3.2 (0.7-10)	3.0 (1-6)	30 (20-55)

Table 3 presents the ESD (mGy) values for both genders and all patients. Table 4 shows the ESD (µGy) for both gastroenterologist and assistant. Table 5 shows the estimated organ dose using the conversion factors from NRPB,[19] risk factors from ICRP[5,6] and the estimated risk values. This Table also provides an estimation of the cancer risks associated with the organ dose. The risk of radiation-induced cancer for different organs was in the magnitude of 10 ^-5^ and 10 ^-6^ per procedure, while the annual examiner risk was in the magnitude of 10 ^-3^ and 10 ^-4^.

**Table 3:** Minimum, median, mean, standard deviation (SD) third quartile and maximum values of ESD. The mean and range of TSD and patient radiation doses (mGy)

Patient dose (mGy)	No.	Mean (±SD)	Minimum	Median	Third quartile	Maximum
ESD	57	75.6±76	9.86	44.79	86.10	268.1
Males	32	73.9±68	10.17	36.77	83.49	268.1
Females	25	77.4±79	9.86	52.81	88.81	260.1
Exit dose	57	3.22±0.26	0.18	1.12	3.92	45.12
Males	32	2.33±0.23	0.19	1.14	4.36	45.12
Females	25	4.06±0.24	0.18	2.10	3.48	44.91
TSD	57	0.80±0.61	0.10	0.46	0.89	1.70
Males	32	0.55±0.53	0.06	0.34	0.74	1.56
Females	25	0.82±0.69	0.10	0.57	1.04	1.70

ESD: Entrance skin dose; TSD: Thyroid surface dose

**Table 4:** Minimum, mean ± SD, median, third quartile and maximum values of staff radiation doses (µGy)

Gastroenterologist	Mean ± SD	Minimum	Median	Third quartile	Maximum
Chest	6.2±2.2	0.2	3.6	6.6	32.5
Thyroid	5.40±0.9	0.2	2.9	5.6	27.6
Forehead	3.81±2.1	0.2	2.4	6.6	26.3
Hand	27.2±46	1.02	13.8	53.1	223.2
Effective dose (µSv)	0. 4±1.7	0.01	0.3	0.5	11.8
**Assistant**					
Chest	0.5±1.8	3.3	2.03	3.3	17.3
Hand	0.2±1.6	6.6	3.1	11.6	32.5
Effective dose (µSv)	0.01±1.2	0.2	0.2	0.2	1.2

**Table 5:** Mean organ radiation equivalent dose (mSv), risk coeffi cients and radiation risk per ERCP procedure

Organ	Organ equivalent dose (mSv)	Nominal risk coeffi cient × 10^-4^Sv^-1^	Radiationinduced cancer probability × 10^-6^
Ovaries	1.29	16	2
Uterus	1.42	6.3	1
Breast	0.28	116	3
Skin	1.91	670	128
Hereditary effect*	1.29	20	3
Effective dose	3.44	550	190

* Nominal risk in the whole population; ERCP: Endoscopic retrograde cholangiopancreatography

## DISCUSSION

### Patient body characteristic data and exposure factors

The patient body characteristic data were comparable to the mean values reported in the literature[7,8,11,12] and these values were higher compared to those of the NRPB standard phantom.[19] In general, variations in BMI and exposure factors influence the patient dose and image contrast.

The mean screening time in the present study is 2.9 min, which was less than that previously reported, which ranged between 6 and 14 min.[8,9,12,13] It is important to note that; Larkin *et al*,[8] and Buls *et al*,[13] estimated that fluoroscopic exposure contributes 90% of patient total dose during ERCP. In agreement with the aforementioned studies, strong correlation was found between the ESD and screening time (*R*^2^ =0.91). Therefore, fluoroscopic time can be a good indicator of dose if radiographic images are controlled. Uradomo *et al*,[16] achieved a reduction in screening time of 10% with pulsed fluoroscopy (5.2 min), which was adjusted to terminate the exposure in 3 sec, compared to continuous fluoroscopy (4.7 min). Unfortunately, they did not report the number of films taken.

The exposure factors (kVp, mA) were comparable to exposure factors reported in previous studies.[8,9,12,13] In general, high kVp increases the scatter radiation thus also the examiners dose, while decreasing the contrast of the image.[24,25] Consequently, Heyd *et al*,[11] reported that, a high kVp technique (80 kVp -100 kVp) could reduce the dose to a patient up to 50%, compared to the conventional technique (75 kVp -96 kVp). The mean number of radiographs in this study was 2.6 per procedure, which is also lower than that in previous studies.[7,8,11–13] As expected, no significant correlation was found between patient dose, patient characteristics, and exposure factors, but it should be noted that dose does depend on the complexity of the procedure. Storing fluoroscopic images could reduce the number of radiographs; this may decrease the image quality but not the yield of the procedure. Selection of the low dose fluoroscopic mode and good patient positioning prior to the procedure could also reduce the radiation dose. Moreover, adequate filtration of an X-ray beam can substantially reduce patient dose up to 70% by improving the quality of the beam while largely maintaining adequate image quality.[24] In comparison with previous studies, the filtration of our machine (4.0 mm Al) was higher than that used in other studies (Buls *et al*,[13] 2.9 mm AL and Tsalafoutas *et al*,[9] 3.5 mm Al). Therefore, a significant radiation dose reduction can be achieved by optimizing the aforementioned factors.

### Patient absorbed and effective doses

In this study, the mean ESD, resulting from ERCP procedure has been estimated to be 75.6 mGy for the total patient population [Table 3]. The mean ESD result was significantly lower compared to previous studies for therapeutic ERCP [Table 6]. However, the majority of the previous studies were mainly for dose survey[7,8,12] and examiner protection.[10–12] Heyd *et al*,[11] reported the highest ESD (733 mGy), number of radiographs (16 radiographs) and screening time (14 min). Our results are 60% lower than the lowest value (178.9 mGy)[13] of the reported studies. In addition, continuous considerable radiation dose reduction was observed concerning the recent published studies compared to the previous ones. This result indicates that a high degree of patient dose reduction was achieved in the present study. This could be attributed to the presence of two experienced gastroenterologists, offering a quick interpretation and decision-making and the low screening time, fluoroscopic captured images and radiographs reduction. Interpretation of image can produce a dilemma for the first examiner whose attention is divided between endoscopic and fluoroscopic monitors and patient[25] The first examiner always concentrates on the endoscopic screen, while the second one monitors the fluoroscopic screen and controls the radiation. Conversely, Buls *et al*,[13] reported that the communication problem between the gastroenterologist and the radiographer affects the radiation dose.

**Table 6:** The mean patient parameters, screening time, number of radiographic images, ESD and effective dose in various therapeutic studies

Author	n	Age (year)	BMI (Kg/m^2^)	Screening time (min)	No. of radiographic images	DAP Gy.cm^2^	ESD (mGy)	Effective dose (mSv)
Sulieman *et al*.	57	66.8 (26-91)	27.3 (17.9-42.9)	2.9 (0.3-12.3)	2.6 (1-6)	NR	75.6	4.16
Larkin *et al*.[8]	12	74.8 (60-89)	NR	10.5 (5.9-16.6)	3.7	66.8	NR	12.4
Tsalafoutas *et al*.[9]	21	66 (34-92)	NR	6 (1.3-23.5)	2.9 (2-4)	41.8	178.9	8.7
Heyd *et al*.[11]*	72	53.6 (20.6-86.5)	26.11 (17.5-61)	14 (2-63)	16 (6-45)	NR	80	NR
Chen *et al*.[12]	12	60 (22-89)	NR	5.9	4	NR	262	NR
Buls *et al*.[13]	54	66.5 (41.5-81)	NR	6	4	49.9	347	9.9

NR: not reported, ESD: Entrance skin dose; DAP: Dose area product,

* Diagnostic procedures, Range is in parenthesis

The mean value of the effective dose was estimated to be 3.85 mSv. Comparison between effective doses from previous studies shows that these values are also lower as shown in Table 6.

As ERCP involves direct irradiation of some of the internal and radiosensitive organs, equivalent doses for specific organs were estimated as illustrated [Table 6]. In comparison with Buls *et al*,[12] the organ radiation doses were also significantly lower. Thyroid surface dose (TSD) was estimated by direct placement of the TLDs to organ site. Ovary dose, which has special concern due to the hereditary effect of radiation, was estimated at 1.29 mGy. The exit dose value was much higher than estimated dose values for the breast, while it was slightly higher than total skin dose.

**Figure 2:**
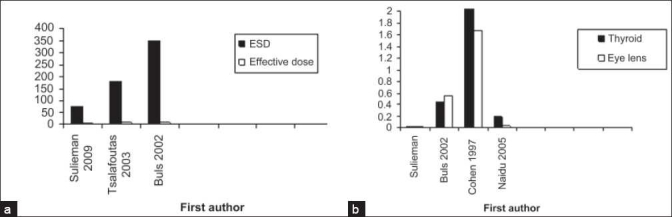
(a) A comparison of ESD and effective dose for ERCP patients with those published, previously. (b) A comparison of thyroid and eye lens doses for staff with previous studies

Previous authors have used different values of conversion factors to derive effective dose from ESD. Buls *et al*,[13] (0.03 mSv.mGy ^-1^) used NRPB software[19] whereas Tsalafoutas *et al*,[8] (0.05 mSv.mGy ^-1^) used a mean value derived from the conversion factors between the two studies,[8,13] which are similar to our result. A comparison of ESD and effective dose for ERCP patients with those published previously, are shown in Figure 2a. These variations could be attributed to the X-ray machine characteristics and to projections used to derive the effective dose from ESD. However, it offers a good and simple indicator of effective dose estimation.

This variation of patient dose is due to differences in the protocols and exposure factors, X-ray equipment, patient pathology, field of view, geometry and examiners experience.

Our study protocol was designed to use intermittent fluoroscopy with fluoroscopic image with last image hold to minimize exposures. Even if these images generally have inferior image quality compared with radiography, it has the required findings. This reduction also reduces the risk of the tissue reactions and offer further margins for further investigations and follows up especially for young and pregnant patients.

### Staff doses

The measured examiners doses (*µ*Gy) are comparable as presented in Table 4. As expected, the first examiner was more exposed than the assistant. The use of full wrap-around apron is more effective due to the full coverage of the back and waist area, than the frontal protection one.

The mean radiation dose to the unprotected parts of the gastroenterologist was higher for the hand (223.2 *µ*Gy) due to the scatter radiation from the protective barrier (lead apron). However, in current study the effective dose per procedure was estimated as 2.04 *µ*Sv and 1.0 *µ*Sv for the gastroenterologist and assistant, respectively. Comparisons of thyroid and eye lens doses for staff with previous studies are shown in Figure 2b.

Dose differences to examiners can be explained in the light of patient dose differences and examiners’ location and the utility of radiation barriers. Regarding the dose limits for workers, examiners can perform over 1000 procedures annually without exceeding the limits (20 mSv per year).[5] However, due to the many variables involved, these results are institution specific and may not be reflective of other institutions.

### Cancer risk estimation

The probability of cancer due to radiation dose depends on organ dose, age and tissue weighting factor, which represent the relative contribution of that organ or tissue to these effects. Radiation-induced cancer probability is shown in Table 5.

The patient radiation risk estimation for fatal cancer per procedure was found to be 19 × 10^-5^ while the female hereditary risk was estimated to be negligible (1 × 10^-8^). Larkin *et al*,[8] and Naidu *et al*,[10] estimated the risk of radiation-induced cancer between 1 in 1700 (3 × 10^-4^) and 1 in 3500 (6 × 10^-4^) per procedure. The annual radiogenic risk to examiners in this study (500 procedures/year) was estimated to be 56 × 10^-6^, 28 × 10^-6^ and 196 × 10^-6^ for the first, second and third examiners, respectively. Since the procedure is obviously justified, the risk of cancer is used as an indicator of the radiation detriment.

Radiation dose reduction can be achieved by wearing the protective eyeglasses and thyroid shields that significantly attenuate scatter radiation. However, the examiners believe that the use of lead glasses impairs vision and so increases the exposure time and that lead gloves are inconvenient for the first and the second examiners.

### Reference dose level

The high doses incurred in interventional radiology procedures make it advisable to establish reference dose values.[26,27] However, reference dose for therapeutic ERCP is complicated because the duration and complexity of the fluoroscopic exposure is strongly dependent on the individual clinical conditions. i.e. the procedure is clinically open-ended, continuing until the procedure is complete.[27]

Therefore, we propose a local reference dose level by using the third quartile value (86.81 mGy associated with 2 radiographs and 3.5 min screening time) as shown in Tables 2 and 4, as a first step towards dose optimization. The available data is still not enough to establish national reference levels, but this could be a baseline for further studies concerning the optimization of dose with regard to avoiding unnecessary radiation risks.

## CONCLUSION

ERCP with fluoroscopic technique demonstrates improved dose reduction by a factor of 2, without compromising the diagnostic findings. The radiation dose to the examiners is well within established safety limits, in the light of the current practice. The radiation absorbed doses to the different organs are relatively low. Furthermore, the examiner should put on a lead wrap-around protective apron, since he is not facing the scattered radiation.
